# Focal liver lesions: multiparametric microvasculature characterization *via* super-resolution ultrasound imaging

**DOI:** 10.1186/s41747-024-00540-3

**Published:** 2024-12-05

**Authors:** Qian-Qian Zeng, Shi-Zhe An, Chao-Nan Chen, Zhen Wang, Jia-Cheng Liu, Ming-Xi Wan, Yu-Jin Zong, Xiao-Hua Jian, Jie Yu, Ping Liang

**Affiliations:** 1 Department of Interventional Ultrasound, Senior Department of Oncology, The Fifth Medical Center of PLA General Hospital, Fengtai District Beijing, 100853 China; 2 Chinese People’s Liberation Army (PLA) Medical School, Haidian District Beijing, 100853 China; 3 The Key Laboratory of Biomedical Information Engineering of Ministry of Education, Department of Biomedical Engineering, School of Life Science and Technology, Xi’an Jiaotong University, Xi’an, 71000 China; 4 School of Materials Science and Intelligent Engineering, Nanjing University, Suzhou, 215163 China

**Keywords:** Carcinoma (hepatocellular), Focal nodular hyperplasia, Liver neoplasms, Microbubbles, Ultrasonography

## Abstract

**Background:**

Noninvasive and functional imaging of the focal liver lesion (FLL) vasculature at microscopic scales is clinically challenging. We investigated the feasibility of using super-resolution ultrasound (SR-US) imaging for visualizing and quantifying the microvasculature of intraparenchymal FLLs.

**Methods:**

Patients with FLLs between June 2022 and February 2023 were prospectively screened. Following bolus injection of microbubbles at clinical concentration, SR-US was performed using a high frame rate (350–500 Hz) modified ultrasound scanner and a convex array transducer with a central frequency of 3.1 MHz.

**Results:**

In total, 47 pathologically proven FLLs at a depth of 5.7 ± 1.7 cm (mean ± standard deviation) were included: 30 hepatocellular carcinomas (HCC), 11 liver metastases (LM), and 6 focal nodular hyperplasias (FNH). The smallest detectable vessel size of the hepatic microvasculature was 128.4 ± 18.6 μm (mean ± standard deviation) at a depth of 8 cm. Significant differences were observed among the three types of lesions in terms of pattern categories, vessel density, minimum flow velocity, and perfusion index. We observed higher vessel density for FNH *versus* liver parenchyma (*p* < 0.001) as well as fractal dimension and local flow direction entropy value for FNH *versus* HCC (*p* = 0.002 and *p* < 0.001, respectively) and for FNH *versus* LM (*p* = 0.006 and *p* = 0.002, respectively).

**Conclusion:**

Multiparametric SR-US showed that these three pathological types of FLLs have specific microvascular phenotypes. Vessel density, fractal dimension and local flow direction entropy served as valuable parameters in distinguishing between benign and malignant FLLs.

**Trial registration:**

ClinicalTrials.gov (NCT06018142).

**Relevance statement:**

Multiparametric SR-US imaging offers precise morphological and functional assessment of the microvasculature of intraparenchymal focal liver lesions, providing insights into tumor heterogeneity and angiogenesis.

**Key Points:**

- Super-resolution (SR)-US imaging allowed morphological and functional evaluation of intraparenchymal hepatic lesion microvasculature.
- Hepatocellular carcinoma, liver metastasis, and focal nodular hyperplasia exhibit distinct microvascular architectures and hemodynamic profiles.
- Multiparametric microvasculature characterization *via* SR-US imaging facilitates the differentiation between benign and malignant microvascular phenotypes.

**Graphical Abstract:**

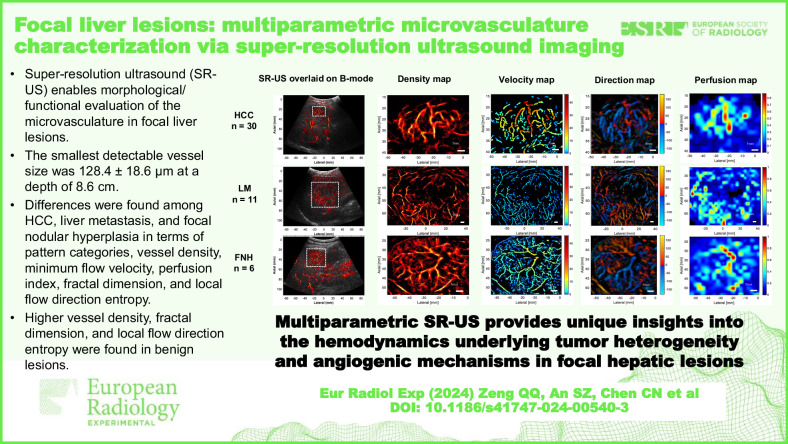

## Background

It is well-accepted that tumor angiogenesis results in an aberrant vascular architecture and functional abnormalities and is associated with tumorigenesis, tumor propagation and progression. Tumor heterogeneity leads to heterogeneity in the tumor microvasculature [1, 2]. Accordingly, structural and hemodynamic information about the vasculature at the microscopic scale has great implications for tumor management in terms of differential diagnosis, understanding tumor biological and pathological characteristics, and identifying subtle changes for evaluating and predicting therapy responses.

Focal liver lesions (FLLs) describe a wide variety of benign and low- or high-grade malignant disorders with varied prognoses and treatment strategies. Therefore, medical imaging approaches that can help obtain morphological and functional parameters of hepatic vascularization are urgently needed and continue to be the focus of research [3–6].

Ultrasound is the most frequently used medical imaging modality in clinical practice because of its safety, ease of accessibility, and high spatial and temporal resolution. Intravenously administered clinically approved ultrasound contrast agents, inert gas microbubbles, function as intravascular acoustic sources due to the impedance mismatch between blood and gas, as well as through nonlinear oscillation at certain acoustic pressure thresholds. These properties greatly enhance the visualization of the vascular bed. Furthermore, by isolating, localizing and tracking microbubbles from many continuously recorded frames in the same imaging plane, super-resolution ultrasound (SR-US) imaging—derived from the foundational concept of ultrasound localization microscopy—has surpassed the classical diffraction limit for ultrasound imaging. This advancement has enabled the observation of subwavelength structural details of the microvascular network and the detection of microregional hemodynamics at the millimeter-per-second scale [7–9].

Most experimental studies implemented SR-US on chicken embryos and rodents [9–14]. Several studies have applied SR-US in clinical pilot studies for imaging the carotid artery, lower limb, renal graft in the iliac fossa, human brain, and some tumors in superficial locations (*e.g*., breast cancer, prostate cancer, pancreatic tumor) [15–21]. However, translating SR-US imaging to clinical applications for managing liver lesions is challenging. Conventional low frame rate (approximately 10–15 Hz) contrast-enhanced ultrasound (CEUS) scanning with clinical scanners has difficulty accurately localizing and tracking fast-moving microbubbles at standard clinical relatively dense concentrations. Using highly diluted microbubbles was proven to be a viable solution [7, 18, 22], but the resulting longer data acquisition time (from dozens of seconds to several minutes) can potentially induce motion interference and increase the difficulty of freehand scanning, which hampers its use in organs with distinct tissue motion patterns, such as the liver.

Ultrafast high frame rate (approximately 500 Hz) SR-US has been reported to successfully image the human kidney and liver with short acquisition times (several seconds). However, the linear array scanning mode used in this previous study was limited by the penetration depth, and it was difficult to achieve deep tissue imaging [20]. Demené et al [18] applied SR-US for transcranial adult vascular imaging with an ultrafast frame rate (800 Hz) and a phased array ultrasonic probe and achieved an imaging depth of more than 10 cm for the first time. However, the collection time was approximately 2 min due to dilution of the microbubbles.

To achieve micron-scale resolution of the hepatic microvasculature in clinical settings, a convex array transducer might be suitable due to its relatively wide curved array window and deep penetrability at low frequencies. No previous reports have attempted to implement ultrafast high frame rates with convex array transducers in SR-US.

In this paper, we present the SR-US imaging of human livers *via* the use of a modified ultrafast frame rate clinical scanner and a convex array transducer, and assessed its feasibility in visualizing and quantifying the microvasculature in FLLs.

## Methods

### Ethics

Our study was registered on ClinicalTrials.gov (NCT06018142), and the study protocol was approved by the Institutional Review Board. Written informed consent was obtained from all enrolled patients.

### Patients

Between June 2022 and February 2023, patients who underwent ultrasound examinations revealing FLLs with a maximum lesion diameter ≥ 1 cm were prospectively screened for inclusion in this study in our department. The exclusion criteria were as follows: (1) previous anti-angiogenic therapy, chemotherapy or locoregional treatment for the target lesion; (2) a poor sonographic window (blocked by lung gas or rib shadow); (3) lesion deeply located (> 10 cm from the skin). Upon inclusion, patients were required to perform the SR-US examinations during the same session. During data postprocessing, patients with (4) unavailable histopathological results, (5) uncorrectable motion interference, or (6) an unidentifiable target lesion were also excluded.

### SR-US imaging settings, data acquisition, and processing

**Fig. 1:**
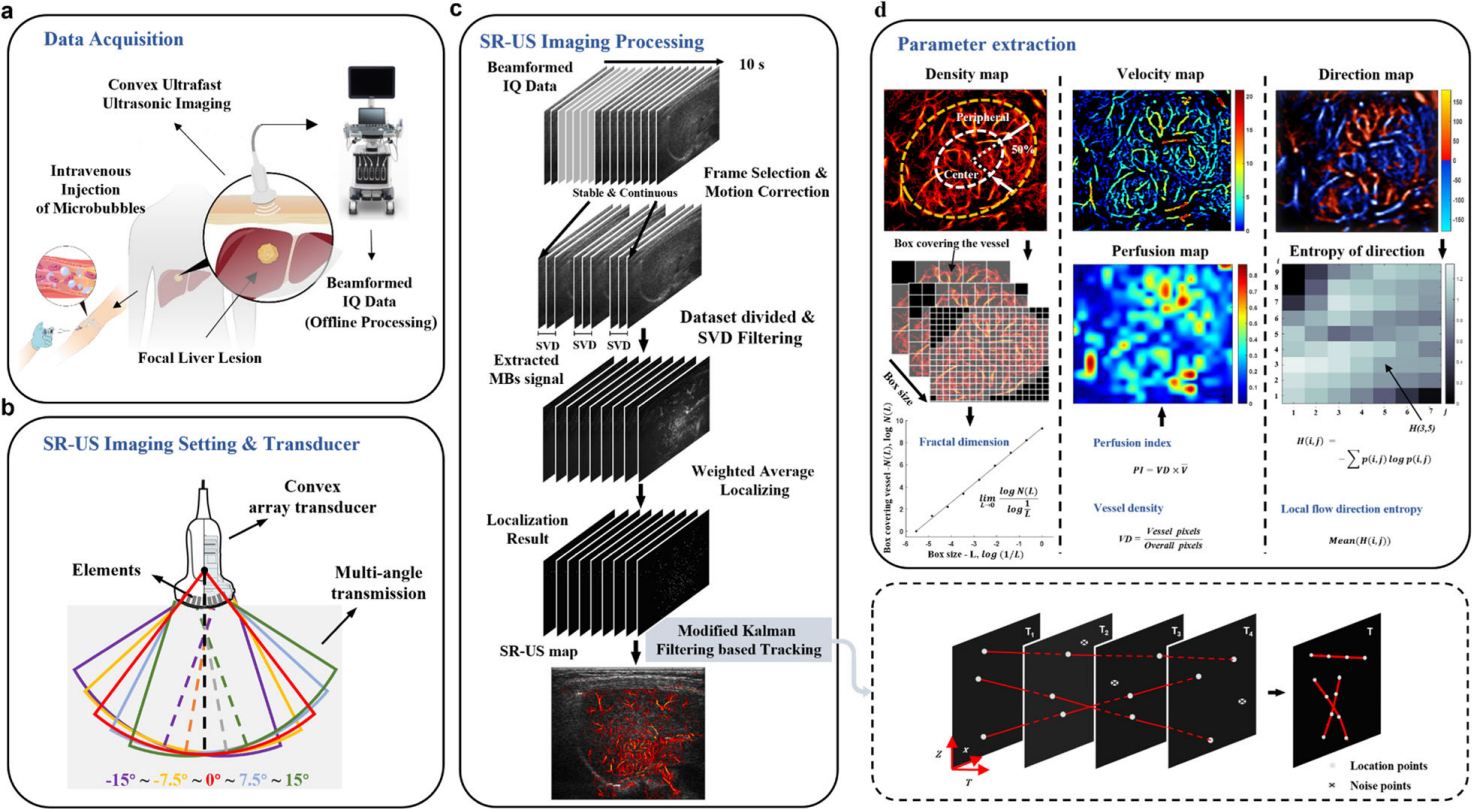
Procedure demonstration of offline super-resolution ultrasound data acquisition, processing and parameter extraction. **a** Data acquisition. **b** Settings and convex array transducer parameters. **c** Imaging processing. Beamformed in-phase/quadrature (IQ) data was selected based on the motion amplitude and corrected by matrix transformation. Then microbubbles signals were extracted through singular value decomposition filtering and located by weighted average. The modified Kalman filter method and interpolation were used to track and rebuild the microbubbles trajectory to generate density maps. Modified Kalman filter-based tracking is displayed in the bottom right. **d** Parameter extraction processing. Yellow dashed line indicates the lesion boundary corresponding to B-mode image. By shrinking the radius inward (the distance between multiple points on the boundary to the center) inward by 50% (white dashed line), each lesion could be divided into central and peripheral regions, the latter defined as the outer 50%; removing the peripheral area yielded the center regions

A modified clinical ultrasound scanner (Resona 7, Mindray, Shenzhen, China) equipped with a convex array transducer (SC6-1U, Mindray, 1.2–5 MHz) was used for SR-US imaging. An ultrafast imaging sequence (350–500 Hz) of diverging waves was designed for the convex array transducer. Multiangle coherently compounded and beamformed in-phase/quadrature data were recorded. (Fig. 1b). After a bolus injection of 1.5 mL of contrast microbubbles (SonoVue, Bracco, Milan, Italy) followed by flushing with 5 mL of normal saline, 3,500–5,000 frames consecutive in-phase/quadrature data were recorded from the first 10 s (to ensure sufficient microbubbles counts for generating the microvascular architecture [23] and clinical feasibility) of the arterial phase. The concentration of microbubbles was set in accordance with the guidelines for clinical CEUS in FLLs (Fig. 1a) [24]. The acquired in-phase/quadrature data were saved and sent for postprocessing in MATLAB (MathWorks Inc., Natick, MA, USA) (Fig. 1c). Settings, acquisition protocol, motion correction, microbubbles isolation, localization, tracking, mapping and contrast-enhanced power Doppler (CE-PD) processing is detailed in Supplementary material.

### SR-US parameter maps and qualitative analysis

**Fig. 2:**
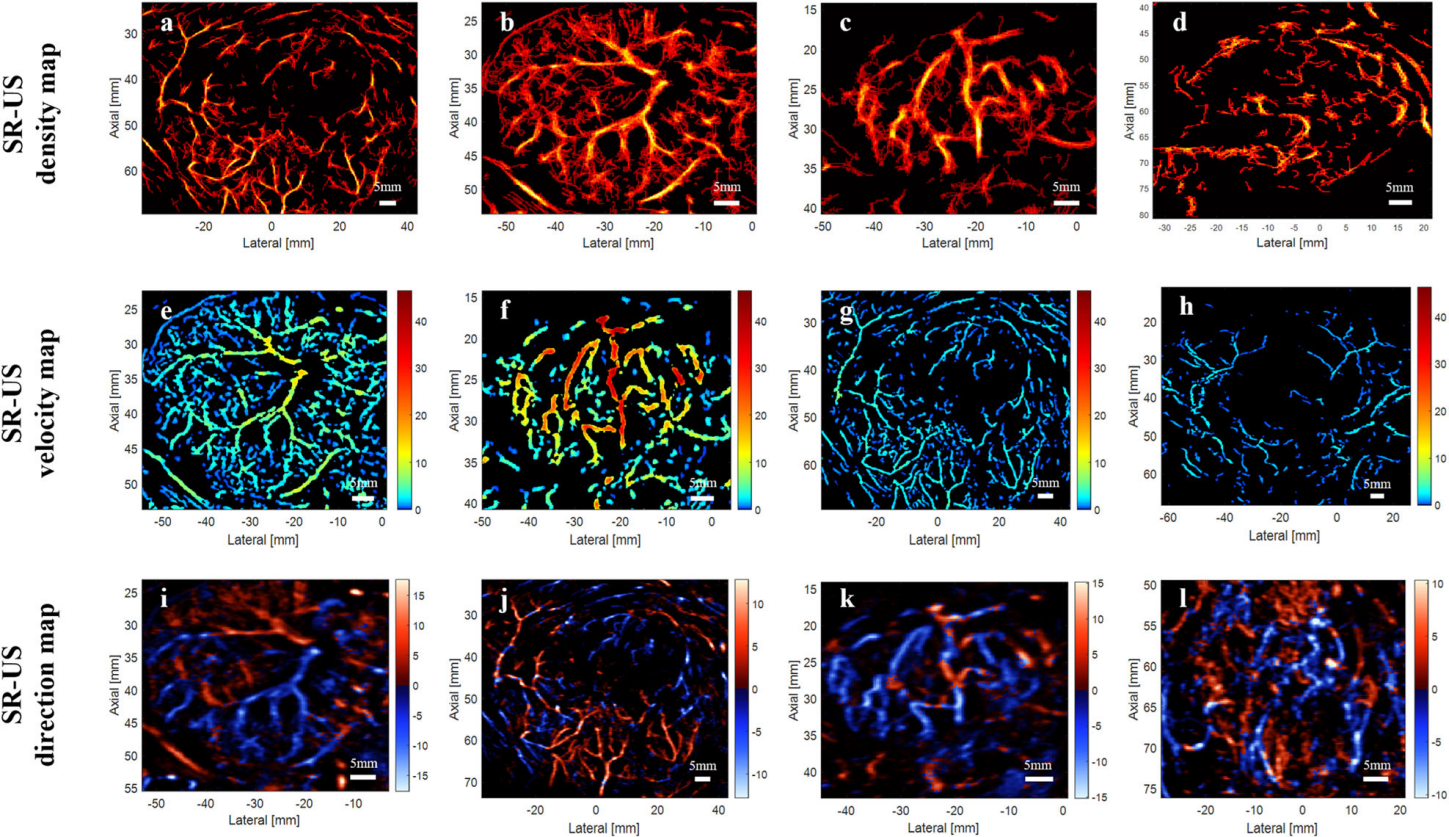
Examples of different super-resolution ultrasound (SR-US) parametric patterns. Density maps: **a** peripherally distributed pattern; **b** well-distributed pattern; **c**, **d** irregularly distributed pattern. The color bar corresponds to the normalized number of localized microbubbles. Velocity maps: **e**, **f** high-speed feeding pattern; **g**, **h** low-speed supplying pattern. The color bar corresponds to different speeds. Direction maps: **i** centrifugal pattern; **j** centripetal pattern; **k** eccentric pattern; **l** mixed pattern. The color bar represents the blood flow direction; red and blue indicate flow toward and away from the transducer, respectively. The white horizontal scale bar on all images represents 5 mm. **a**, **g** and **j** are images of a 51-year-old female patient with liver metastasis; **b**, **e**, **i** of a 35-year-old male patient with focal nodular hyperplasia; **c**, **f**, **k** of a 71-year-old female patient with hepatocellular carcinoma; **d** of a 65-year-old female patient with hepatocellular carcinoma; **h** of a 45-year-old male patient with liver metastasis; **l** of another 65-year-old female patient with hepatocellular carcinoma

The SR-US velocity map, direction map and perfusion map were derived from encoding and overlying the parameters on the SR-US density map (Fig. 1d). For density maps, microvessels located mainly in the lesion periphery with relatively sparse centers were defined as having a peripherally distributed pattern (Fig. 2a). A well-distributed pattern was defined as vessels displaying an axial symmetric distribution, namely, a uniform distribution throughout each quadrant of the entire lesion (Fig. 2b). High- and low-density areas that were separated and scattered unevenly were classified as an irregularly distributed pattern (Fig. 2c, d). For velocity maps, a high-speed feeding pattern was defined when at least two vessels with distinctly faster blood flow could be observed (Fig. 2e, f). In contrast, the low-speed supplying pattern was defined as the lack of a recognizable higher-speed main trunk or the presence of only a single faster flow trunk (Fig. 2g, h). Based on the overall flow direction, direction maps could be classified as having a centrifugal (Fig. 2i), centripetal (Fig. 2j) pattern, or eccentric pattern (Fig. 2k), which was defined as blood flowing from the edge of the eccentricity of the lesion toward the opposite side, or a mixed pattern (Fig. 2l), which was defined as vessels mingling together without a specific directional trend. All the parameter maps were blindly and visually reviewed by one author (C.N.C., with 10 years of experience with abdominal ultrasound) and then summarized and classified into one of several SR-US patterns depending on the macroscopic parametric features. After training, two other radiologists (Z.W. and Q.Q.Z., with 10 years and 4 years of experience with abdominal ultrasound, respectively) reviewed the images again and assigned all the SR-US parameter maps to those parametric patterns. If there were discrepancies between the two readers, the images were reevaluated together by a third, more experienced radiologist (Y.J., with 17 years of experience with abdominal ultrasound) until a consensus was reached. As perfusion maps were generated by the combination of density and velocity measurements, no further analysis was devoted to defining the perfusion patterns.

### SR-US quantitative analysis

The microvascular quantitative parameters analyzed in this paper included: (1) vessel diameter, indicated by the full width at half-maximum of the amplitude distribution of the Gaussian fit curve; (2) density-relevant parameters, calculated from the labeled pixels; (3) velocity-relevant parameters calculated from the microbubble motion trajectory; (4) perfusion-relevant parameters, calculated by mean velocity multiplied by density; (5) fractal dimension of the tumor vasculature, calculated by the box-counting algorithm on the SR-US density maps; and (6) local flow direction entropy, defined as information entropy extraction of flow direction. All the methodologies used are detailed in the Supplementary material.

### Statistical analysis

Statistical analyses were performed using SPSS (version 22, IBM, Armonk, NY, USA) and GraphPad Prism 9.5.1 (GraphPad Software, San Diego, CA, USA). Categorical variables are presented as counts with percentages. Quantitative parameters were presented as medians with interquartile ranges. The rest of the continuous variables were presented as means and standard deviations with minimum-maximum ranges. The Cohen κ with 95% confidence interval was applied to measure the inter-reader agreement between two readers. (poor, 0–0.20; fair, 0.21–0.40; moderate, 0.41–0.60; good, 0.61–0.80; or excellent, 0.81–1.00). The correlation between FLL groups and SR-US parameter pattern discrimination was validated using a χ^2^ test. The *p*-value was subsequently adjusted using Yates’ correction for continuity and the Fisher exact test, contingent upon the statistical data volume and expected frequency. Specifically, Yates’ correction for continuity was applied when the sample size (*n*) ≥ 40 and the expected frequency T ranged 1 ≤ T ≤ 5. Conversely, the Fisher exact test was employed when *n* ≤ 40. The parameter differences between FLL groups were evaluated with the one-way ANOVA. For controlling the false discovery rate caused by multiple comparisons, Bonferroni *post hoc* tests for data with homogeneity variance and Games-Howell tests for data with non-homogeneity variance were applied. Pairwise comparisons regarding vascular density of FLLs and their liver parenchymal were calculated by *t*-test. All presented *p*-values were adjusted (two-sided), and *p* < 0.050 was considered to be statistically significant.

## Results

### Patient and lesion characteristics

**Fig. 3:**
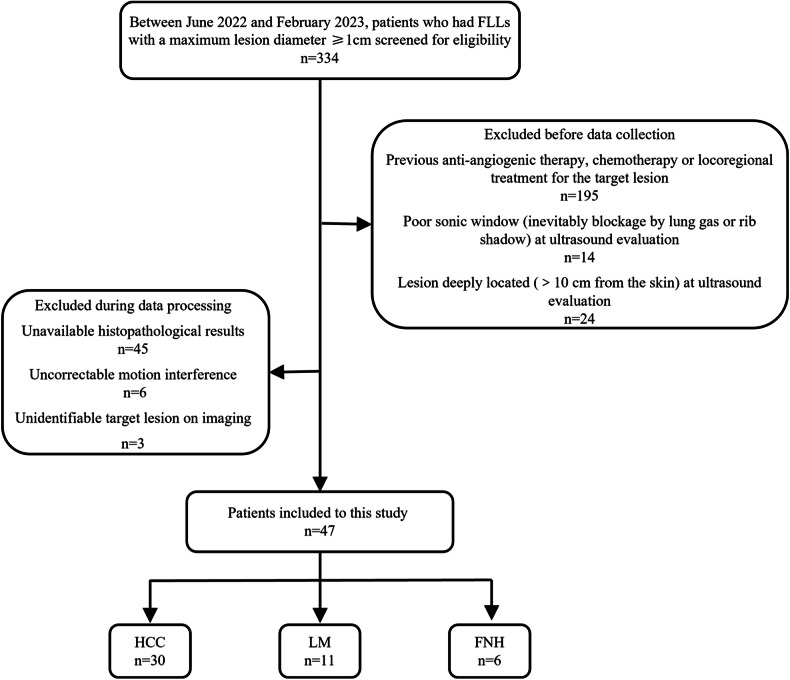
Flowchart showing study inclusion and exclusion criteria. FLLs, Focal liver lesions; FNH, Focal nodular hyperplasia; HCC, Hepatocellular carcinoma; LM, Liver metastasis

A total of 47 patients (aged 58.5 ± 15.0 years, mean ± standard deviation; 34 males) were recruited (Fig. 3). The baseline characteristics of the patients and FLLs are presented in Table 1. The mean body mass index was 23.9 ± 3.3 kg/m^2^ (range 17.2–28.6 kg/m^2^). The mean size of the FLLs was 3.3 ± 1.2 cm (range 1.4–7.1 cm). The mean depth from the liver capsule was 5.7 ± 1.7 cm (range 2.6–8.6 cm). Pathology revealed that all the FLLs could be divided into hepatocellular carcinoma (HCC, 30/47, 63.8%), liver metastasis (LM, 11/47, 23.4%) and focal nodular hyperplasia (FNH, 6/47, 12.8%).

**Table 1:** Baseline characteristics of 47 patients and FLLs

Characteristic	Value
Gender (*n*, %)	
Male	34 (72.3)
Age (years)	58.5 ± 15.0 (28–90)
Body mass index (kg/m^2^)	23.9 ± 3.3 (17.2–28.6)
Hepatic steatosis (*n*, %)	24 (51.1)
Size of FLLs (cm)	3.3 ± 1.2 (1.4–7.1)
Depth of FLLs (cm)	5.7 ± 1.7 (2.6–8.6)
Pathological diagnosis (*n*, %)	
Hepatocellular carcinoma	30 (63.8)
Liver metastasis	11 (23.4)
Focal nodular hyperplasia	6 (12.8)

Continuous variables are presented as mean ± standard deviation, with ranges in parentheses. Categorical variables are the counts with percentages in parentheses. Depth of FLLs indicates the distance from the skin to the farthest posterior edge of the lesion at B-mode US

*FLLs* Focal liver lesions

### SR-US image and quantification of resolution

**Fig. 4:**
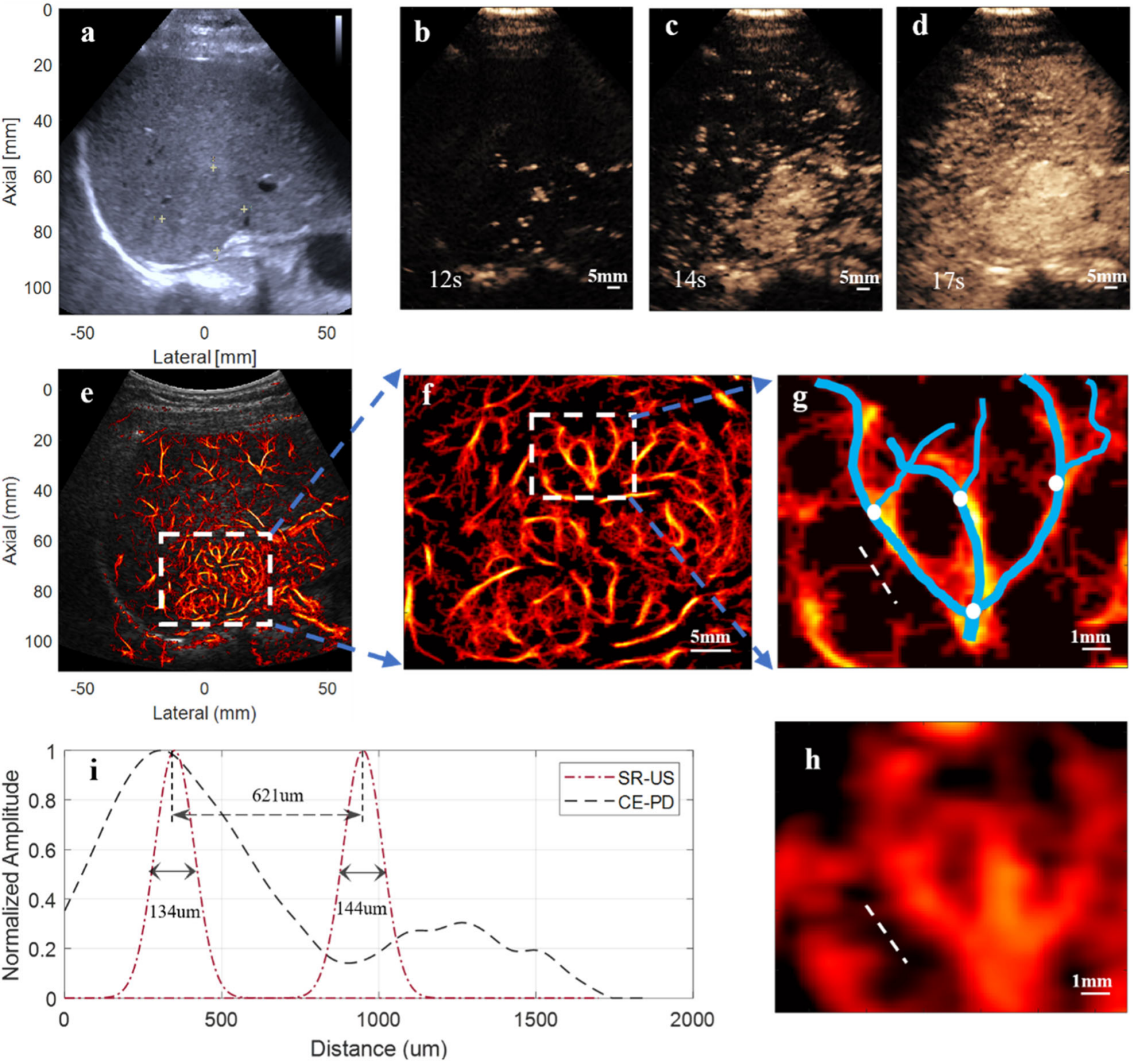
Focal nodular hyperplasia in a 38-year-old female patient. **a** B-mode image. **b**–**d** Contrast-enhanced ultrasound image. **e** Super-resolution ultrasound (SR-US) microvascular density image overlaid on the ultrasound B-mode image. **f** The lesion was zoomed-in region as indicated by the white rectangle in **e**. **g** Magnified region indicated by the white rectangle in **f**. **h** Contrast-enhanced power doppler (CE-PD) image of the same region in **g**. **i** Vessel cross-section intensity profile of SR-US and CE-PD image. Intensity profile of the vessel cross-section, delineated by the white dashed line in the SR-US image (**g**) and the CE-PD image (**h**), is represented by the red and black dashed curves, respectively

Figure 4 shows an example of the deepest lesion in the study. This FNH lesion is iso-echoic, and the depth of its farthest posterior edge is 8.6 cm on B-mode ultrasound (Fig. 4a). On CEUS images, the lesion shows arterial phase hyperenhancement, characterized by early and greater enhancement in the central region, subsequently followed by peripheral enhancement (Fig. 4b–d). A fine and detailed microvasculature is clearly revealed on SR-US images (Fig. 4e–g), as in the magnified region of interest, the generation of vessel branches and turning points can be resolved (Fig. 4f, g), whereas on the CEUS and CE-PD images, these features cannot be visualized (Fig. 4h). The intensity cross-sectional profiles further illustrate the improvement in resolution of the SR-US image compared with the corresponding CE-PD image (Fig. 4i), in which 134 μm- and 144 μm-wide microvessels can be distinguished on SR-US image (Fig. 4g), but are inseparable on CE-PD image (Fig. 4h). On five different manually selected, cross-sectional regions of interest, the average smallest detectable vessel size was 128.4 ± 18.6 μm on SR-US image, 5.9 to 6.5 times thinner than that measured on CE-PD image (Supplementary Fig. S1, Table S2).

### SR-US parametric patterns of FLLs

The inter-reader agreement of SR-US density, velocity, and direction pattern categories were to be rated as having good, excellent, and good, respectively (Table 2). As illustrated in Table 3, between the three FLL groups, significant differences were observed in density, velocity, and directional patterns, with the exception of the velocity patterns between HCC and FNH (*p* = 0.564). Most HCCs had the irregularly distributed density pattern (63.3%, 19/30), and either the eccentric (60.0%, 18/30) or mixed direction pattern (30.0%, 9/30). Furthermore, more HCCs had the high-speed feeding pattern (83.3%, 25/30) than the low-speed supplying pattern (16.7%, 5/30). The majority of LMs demonstrated peripherally distributed and low-speed supplying patterns (81.8%, 9/11 and 72.7%, 8/11, respectively), with occasional irregularly distributed density patterns observed (9.1%, 1/11), as depicted in Supplementary Fig. S2. Approximately half of the LMs exhibited the centripetal direction pattern (54.5%, 6/11), and the other half exhibited the mixed (36.4%, 4/11) or eccentric pattern (9.1%, 1/11). All FNHs displayed a high-speed feeding pattern (100%, 6/6), with the majority exhibiting well-distributed density and centrifugal direction patterns (83.3%, 5/6 and 66.7%, 4/6, respectively). Additionally, FNHs demonstrated a mixed direction pattern in a subset of cases (33.3%, 2/6), as illustrated in Supplementary Fig. S3. A minority of HCC cases (6.7%, 2/30) displayed characteristics such as a well-distributed density pattern, high-speed supply velocity pattern, and centrifugal direction pattern resembling FNH, as depicted in Supplementary Fig. S4. Additionally, HCC may exhibit a peripherally distributed density pattern (20.0%, 6/30) similar to LM, as illustrated in Supplementary Fig. S5.

**Table 2:** Inter-reader agreement regarding the evaluation of super-resolution ultrasound parametric pattern

Parameter	κ values	Agreement
Density pattern category	0.734 (0.565, 0.903)	Good
Velocity pattern category	0.884 (0.714, 1.055)	Excellent
Direction pattern category	0.718 (0.548, 0.889)	Good

Data in parentheses are 95% confidence intervals

**Table 3:** Frequencies of super-resolution ultrasound parametric patterns in focal liver lesions

Parametric pattern	HCC (*n* = 30)	LM (*n* = 11)	FNH (*n* = 6)	*p-v*alue HCC *versus* FNH	*p-v*alue LM *versus* FNH	*p-v*alue HCC *versus* LM
Density pattern						
Peripherally distributed (*n*, %)	6 (20.0)	9 (81.8)	0 (0)	**0.004**	**< 0.001**	**< 0.001**
Well-distributed (*n*, %)	5 (16.7)	1 (9.1)	5 (83.3)			
Irregularly distributed (*n*, %)	19 (63.3)	1 (9.1)	1 (16.7)			
Velocity pattern						
High-speed feeding (*n*, %)	25 (83.3)	3 (27.3)	6 (100)	0.564	**0.009**	**0.002**
Low-speed supplying (*n*, %)	5 (16.7)	8 (72.7)	0 (0)			
Direction pattern						
Centrifugal (*n*, %)	2 (6.7)	0 (0)	4 (66.7)	**0.002**	**0.002**	**< 0.001**
Centripetal (*n*, %)	1 (3.3)	6 (54.5)	0 (0)			
Eccentric (*n*, %)	18 (60.0)	1 (9.1)	0 (0)			
Mixed (*n*, %)	9 (30.0)	4 (36.4)	2 (33.3)			

Categorical variables are presented as counts with percentages in parentheses. The correlation between FLL groups and parameter pattern discrimination was validated using a χ^2^ test. To adjust *p*-value upon the statistical data volume and expected frequency, Yates’ correction for continuity was applied when the sample size was *n* ≥ 40 and the expected frequency T ranged 1 ≤ T ≤ 5. The Fisher exact test was employed when *n* ≤ 40. *p*-values below the significance threshold (*p* < 0.050) are highlighted in bold

*HCC* Hepatocellular carcinoma, *LM* Liver metastasis, *FNH* Focal nodular hyperplasia

### Visualization of hemodynamic functional information

**Fig. 5:**
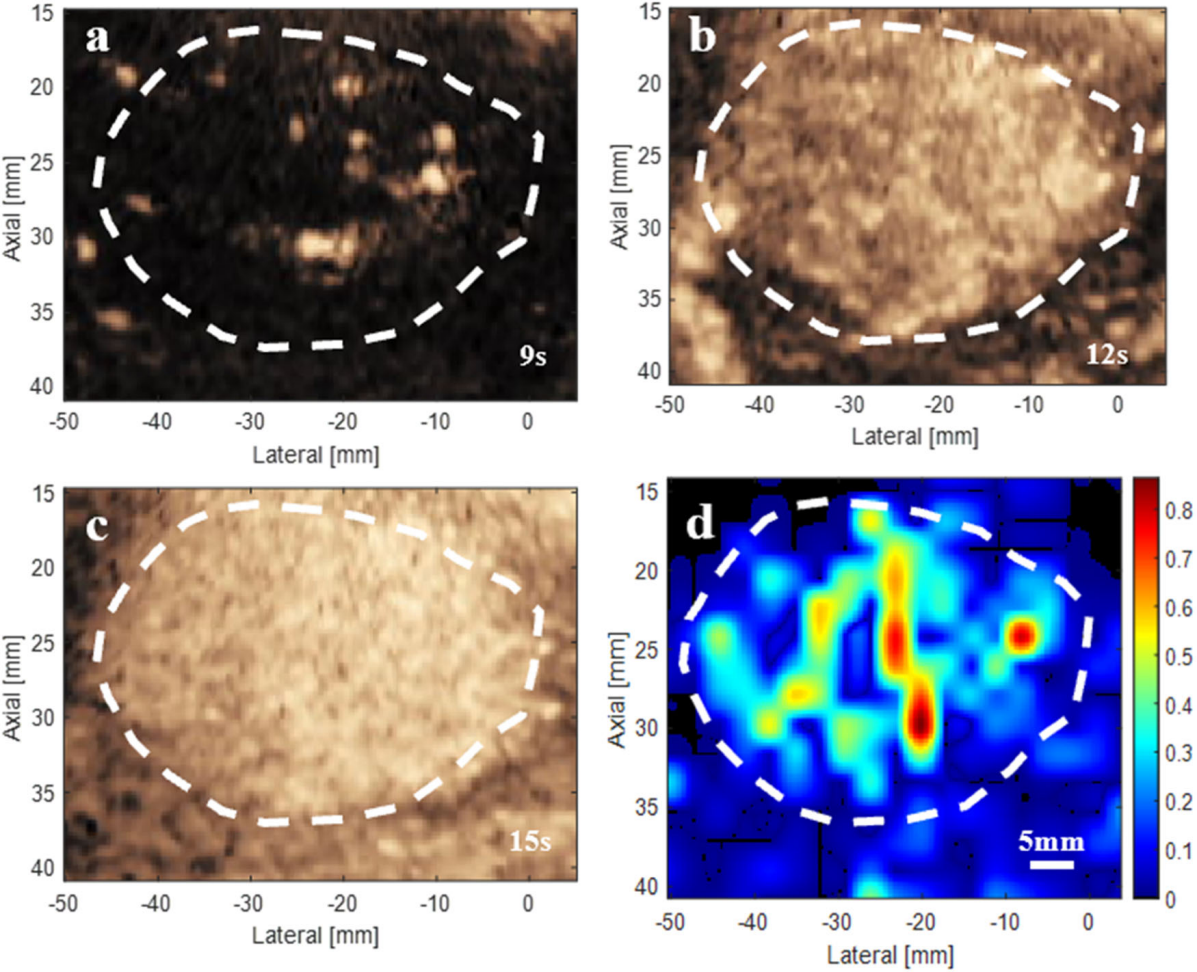
Contrast-enhanced ultrasound (CEUS) image and super-resolution ultrasound (SR-US) perfusion map of a 71-year-old female patient with hepatocellular carcinoma. **a**–**c** CEUS image. **d** The corresponding SR-US perfusion map. The white dashed line indicates the lesion boundary

The SR-US perfusion maps were generated by multiplying the mean velocity by the normalized number of localized microbubbles on individual microvessels. Figure 5 demonstrates the different property of the CEUS and the SR-US images in identifying regional perfusion characteristics. The sequential CEUS images captured over time could dynamically illustrate the temporal distribution of blood flow at specific moments (Fig. 5a–c). While considering data over a range of time points, the SR-US perfusion map enables the quantification and localization of hemodynamic function, revealing heterogeneous distributions of blood perfusion throughout the vascular network of the lesion (Fig. 5d).

### Characterization of FLLs from SR-US quantitative parameters

Table 4 shows the quantitative SR-US parameters of the different lesion types. Regarding the density parameters, the central vessel density was the lowest in the LM group and highest in the FNH group, and this was the only parameter allowed discrimination among the three groups (FNH *versus* HCC, *p* = 0.013; FNH *versus* LM, *p* < 0.001; HCC *versus* LM, *p* < 0.001). Regarding total vessel density and peripheral vessel density, FNH also had higher values than did HCC (total area, *p* < 0.001; peripheral area, *p* < 0.001) and LM (total area, *p* < 0.001; peripheral area, *p* < 0.001), but there was no significant difference between the HCC and LM groups. Regarding the velocity-based parameters, the LM group exhibited a lower minimum flow velocity in the total area than did the FNH group (*p* = 0.038) and a lower minimum flow velocity in the central area than did the HCC group (*p* = 0.049), however, the mean and maximum flow velocities were not significantly different among the three groups. The perfusion-related parameters were derived by combining the density and velocity information; the LM group had a lower perfusion index in the total area than did the HCC group (*p* = 0.049), and a lower perfusion index in the central area than did the FNH group (*p* = 0.009). In terms of the fractal dimension, which represents the structural information of the entire lesion, the FNH group had the highest value, which differed significantly from that of the other two groups (FNH *versus* HCC, *p* = 0.002; FNH *versus* LM, *p* = 0.006); however, no significant difference was found between the HCC group and the LM group. The local flow direction entropy contains both structural and directional information about the entire lesion; the FNH group also had the highest value, which differed significantly from that of the other two groups (FNH *versus* HCC, *p* < 0.001; FNH *versus* LM, *p* = 0.002); however, no significant difference was found between the HCC and LM groups.

**Table 4:** Comparison of super-resolution ultrasound quantitative parameters between different focal liver lesions

Quantitative parameter		HCC (*n* = 30)	LM (*n* = 11)	FNH (*n* = 6)	*p*-value HCC *versus* FNH	*p*-value LM *versus* FNH	*p-v*alue HCC *versus* LM
Density-based							
Vessel density	Total area	0.316 (0.252, 0.396)	0.243 (0.228, 0.292)	0.520 (0.466, 0.544)	**< 0.001**	**< 0.001**	0.195
	Central area	0.336 (0.262, 0.455)	0.139 (0.123, 0.196)	0.506 (0.458, 0.645)	**0.013**	**< 0.001**	**< 0.001**
	Peripheral area	0.317 (0.237, 0.373)	0.273 (0.235, 0.339)	0.504 (0.466, 0.544)	**< 0.001**	**< 0.001**	1.000
Velocity-based							
Mean flow velocity (mm/s)	Total area	5.299 (3.473, 14.422)	4.127 (3.273, 4.494)	8.045 (6.154, 22.786)	0.282	0.064	0.640
	Central area	4.336 (2.933, 14.780)	3.015 (2.077, 4.070)	9.368 (6.830, 20.305)	0.288	0.081	0.787
	Peripheral area	5.616 (3.266, 13.198)	4.125 (3.363, 4.662)	7.883 (5.614, 23.654)	0.291	0.601	0.063
Maximum flow velocity (mm/s)	Total area	16.707 (11.586, 51.974)	14.881 (13.205, 19.180)	28.258 (20.207, 54.948)	1.000	0.469	1.000
	Central area	10.960 (8.232, 35.597)	10.627 (5.561, 15.436)	25.635 (18.896, 38.325)	0.494	0.959	0.175
	Peripheral area	16.707 (11.586, 49.470)	14.881 (13.205, 19.180)	22.433 (18.485, 53.260)	0.769	0.487	0.612
Minimum flow velocity (mm/s)	Total area	0.218 (0.145, 0.364)	0.169 (0.145, 0.205)	0.259 (0.229, 0.587)	0.163	**0.038**	0.665
	Central area	0.301 (0.191, 0.827)	0.205 (0.173, 0.265)	0.346 (0.324, 5.219)	0.506	0.358	**0.049**
	Peripheral area	0.229 (0.181, 0.409)	0.169 (0.145, 0.205)	0.259 (0.229, 0.587)	0.394	0.055	0.365
Both density- and velocity-based							
Perfusion index (mm/s)	Total area	2.126 (0.892, 4.558)	0.884 (0.722, 1.648)	3.992 (3.103, 14.332)	0.359	0.195	**0.049**
	Central area	1.491 (0.813, 4.999)	0.486 (0.259, 0.921)	4.375 (3.267, 15.627)	0.066	**0.009**	0.421
	Peripheral area	2.057 (0.901, 4.027)	1.050 (0.818, 1.774)	4.146 (2.704, 12.529)	0.369	0.223	0.107
Structure-based							
Fractal dimension		1.562 (1.501, 1.615)	1.566 (1.531, 1.607)	1.641 (1.681, 1.693)	**0.002**	**0.006**	1.000
Both structure- and direction-based							
Local flow direction entropy		0.784 (0.723, 0.879)	0.725 (0.692, 0.799)	0.895 (0.873, 0.917)	**< 0.001**	**0.002**	0.356

Data are medians with interquartile ranges in parentheses. The comparisons between groups were evaluated with the one-way ANOVA, with Bonferroni *post hoc* tests for data with homogeneity variance and Games-Howell tests for data with non-homogeneity variance. All presented *p-*values were adjusted (two-sided), and *p* < 0.05 was considered to be statistically significant. By shrinking the radius inward (the distance between multiple points on the boundary to the center) inward by 50%, each lesion could be divided into central and peripheral regions, the latter defined as the outer 50%; removing the peripheral area yielded the center regions. *p*-values below the significance threshold (*p* < 0.050) are highlighted in bold

*HCC* Hepatocellular carcinoma, *LM* Liver metastasis, *FNH* Focal nodular hyperplasia

**Fig. 6:**
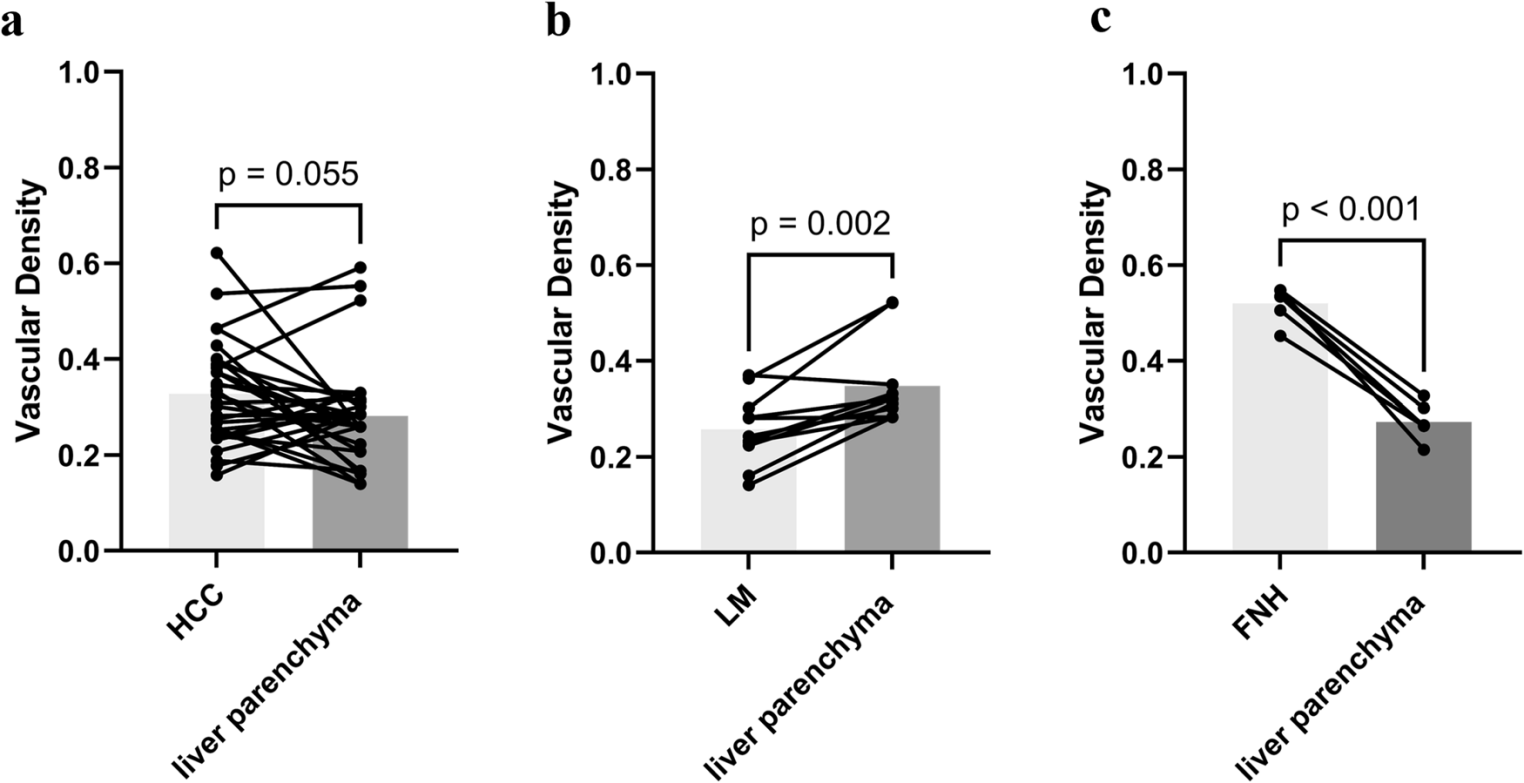
Pairwise comparative analysis of vascular density in focal liver lesions and the surrounding liver parenchyma. **a** Difference between hepatocellular carcinoma (HCC) group and liver parenchyma. **b** Difference between liver metastasis (LM) group and liver parenchyma. **c** Difference between focal nodular hyperplasia (FNH) group and liver parenchyma

The comparative findings of vascular density in FLLs in relation to the background liver parenchyma were illustrated in Fig. 6. Statistical analysis revealed that the vascular density difference between the HCC group and its liver parenchyma was not deemed significant (*p* = 0.055). Conversely, the LM group exhibited lower vascular density compared to its liver parenchyma (*p* = 0.002), while the FNH group demonstrated higher vascular density relative to its liver parenchyma (*p* < 0.001).

## Discussion

By applying a low-frequency convex array probe in a modified commercial Ultrasound system, SR-US imaging was conducted on a population with normal to slightly elevated body mass index. This approach achieved a penetration depth of 8.6 cm, with a detectable microvessel size resolved to 128.4 ± 18.6 μm, providing a field of view comparable to that of conventional ultrasound modalities that was sufficient to observe clinical FLLs in routine patient examinations. A high frame rate and spatiotemporal singular value decomposition-based clutter filter allowed better extraction of microbubbles signals from background tissue at relatively high microbubbles concentrations in a mere 10 s of data, thereby reducing the difficulty of data acquisition and motion correction to some extent. Under high microbubble concentration and intraparenchymal conditions, we applied modified Kalman filtering to better track microbubbles according to existing history trajectories, eliminating the interference of sudden changes in direction and excessive acceleration and yielding more detailed microvascular structures in the internal and deep sections of the lesions. Velocities and vessel diameters over a large range could be obtained simultaneously; up to 0.74–7.94 cm/s for 0.42–0.66 mm-wide vessels and down to 0.07–0.72 mm/s for 98–106 μm-wide microvessels. Also, given the reasonable acquisition time and the convenience of freehand scanning, which obviates the need for complex fixed grippers, coupled with the commercial availability of microbubbles within recommended dosage and administration guidelines for hepatic applications, the standardization and implementation of SR-US imaging protocols could be readily achieved in clinical settings.

SR-US images of FLLs can be evaluated and compared by categorizing microvascular patterns and statistically calculating parameter values. HCCs exhibited the most heterogeneous phenotypes, as all the qualitative patterns could be observed in the HCC group (Table 3), which echoes previous studies on HCC angiogenesis [2, 25, 26]. In contrast, with a blurred distinction between the normal liver parenchyma microvasculature, LMs were more likely to have thin and ramified microvessels distributed at the lesion periphery and exhibit relatively low perfusion characteristics. These findings echo the “rim-like” features of LMs observed with other enhanced imaging modalities [24, 27]. FNH, a benign hepatic tumor, exhibited the highest overall, central and peripheral vascular density values when compared to the HCC and LM groups. Additionally, FNH exhibited greater vascular density than the background liver parenchyma. Previous research suggests that the lower microvascular structure density in HCC compared to normal liver parenchyma, combining the above results may indicate a correlation with benign pathological behaviors characterized by higher vascular density [2, 28]. On the other hand, FNH commonly exhibits early arterial phase enhancement and uniform, centrifugal enhancement, often displaying a “spoke-wheel” pattern and a central feeding artery on CEUS. However, the detection rate of these features varies widely in different studies, ranging from 20 to 90%. This variability may be attributed to the brief observation window, necessitating repeated viewing of video images within a 2-s timeframe, which is highly dependent on the operator’s expertise and the observer’s cognitive abilities. Additionally, discrepancies exist in the literature regarding the classification of the ‘spoke-like’ feature and centrifugal enhancement as either a singular entity or distinct features [29]. Kang et al utilized Doppler-based super-resolution ultrasound, which displayed microvessels by suppressing clutter noises while preserving minimal blood flow signals without the use of a contrast agent, to examine 62 cases of FNH. Their study revealed a 63% detection rate of the ‘spoke sign’ within the lesions, a finding that did not significantly differ from the detection rate observed with CEUS [30]. Most of the FNHs in our study presented with thick and high-velocity vessels generated from the center of the lesion, which then sprouted into secondary and tertiary branches radiating toward the periphery with sharp microvascular margins, leading to a 66.7% (4/6) detection rate for the well-distributed and centrifugal microvascular patterns in the FNH group, which was similar to the detection rate of the ‘spoke sign’ and ‘arterial hyperenhancement’ features observed in other microvascular flow imaging modalities. The remaining FNH lesions (2/6) in this study also exhibited hypervascularity; one displayed a well-distributed vascular network, but the direction patterns of both were mingled and mixed, similar to what has been observed in HCC. This finding also echoes the difficulty in differentiating between HCC and atypical FNH [29–31].

In this study, we analyzed the structural features of SR-US images by applying two established methods [10, 32], obtaining the fractal dimension and local flow direction entropy for each entire lesion area. Notably, FNHs showed higher fractal dimension and local flow direction entropy values than HCCs and LMs, while there was no significant difference between HCCs and LMs (Table 4). Our hypothesis posits that the observed result can be attributed to the presence of a mature vascular architecture in FNHs, characterized by centrally malformed feeder arteries and vascular shunts with hierarchical branches extending from larger blood vessels to well-organized venules and arterioles [31, 33]. It is possible that the coexistence of multiple levels of feeding and draining vessels of different sizes and directions leads to greater structural and direction-based measurements. In contrast, HCCs typically display an aberrantly arterialized and capillarized microvasculature that exhibits irregular sprouts and branches with sudden dead ends or arteriovenous fistulae [1, 2]. This low vascular hierarchy observed in HCC might underlie the observed lower structural parameter value in this study. This finding is consistent with the research conducted by Opacic et al [34], which identified higher local flow direction entropy in malignant tumor models. This suggests that fractal dimension and local flow direction entropy may serve as valuable parameters for distinguishing between benign and malignant FLLs.

Several limitations can be identified in this study. First, as there was a compromise between spatial resolution and acquisition time [22], to ensure clinical feasibility, we sacrificed longer acquisition time, resulting in a resolution that can only resolve the arterioles (20–200 μm) and cannot resolve the capillaries (a few μm). Second, due to the lack of previous research describing the characteristics of microvascular images of focal liver lesions utilizing ultrasound localization microscopy-based technology, and in light of the rigor of our initial depiction and synopsis, our investigation solely encompassed lesions with clear pathological manifestations, thus inadvertently excluding certain diminutive, benign tumors that conventionally necessitate only long-term monitoring, such as hemangiomas, cirrhotic regenerative nodules and dysplastic nodules, leading to selection bias. Therefore, our results here should be considered exploratory. Longer time-span, multidisciplinary, multicenter studies should be conducted to validate the generalizability of the study’s findings. In addition, as our SR-US acquisition protocol was quite harsh owing to ultrasound localization microscopy-based technical limitations, including the need for a proper lesion location, breath-holding coordination and probe maneuvering, etc., certain lesions were likely excluded, thus, there may be representation bias.

In conclusion, three pathologic types of FLLs were translated into clinical practice to serve as a model to evaluate the *in vivo* imaging and differentiation capabilities of SR-US for visualizing hepatic microvascular phenotypes. Furthermore, the use of SR-US parameter maps enhanced the visibility of the functional microvascular networks in FLLs, facilitating the identification of specific microregions of interest for analyzing subtle hemodynamic changes within each lesion. Also, the good-to-excellent repeatability of SR-US imaging and the objectivity of the quantitative parameters of SR-US open a way to perform noninvasive longitudinal and comparative studies on FLLs, which holds significant value in elucidating the fundamental mechanisms of tumor angiogenesis and establishing innovative hemodynamic-related models for assessing and predicting therapeutic responses in oncologic research.

## Supplementary information

**Additional file 1**: **Supplementary Table S1** Modified for the ultrafast imaging mode in Resona 7. **Supplementary Figure S1**: Display the five different manually cross-sectioned ROI of the same SR-US density map with Fig. 4. The vessel size measured as the full width at half-maximum of the amplitude distribution of the Gaussian fit curve on SR-US image and corresponding CE-PD image in each selected ROI. CE-PD = contrast-enhanced power Doppler, SR-US = super-resolution ultrasound, ROI = region of interest. **Supplementary Table S2**: The cross-section measurement of CE-PD mode and SR-US mode in each selected ROI. **Supplementary Figure S2**: SR-US parameter maps of an atypical liver metastasis. (**a**) The SR-US density map indicates that the microvascular structure of the lesion exhibits chaotic distribution throughout the lesion area, displaying an irregularly distributed density pattern. The color bar corresponds to the normalized number of localized microbubbles. (**b**) The SR-US velocity map indicates that the central vascular velocity of the lesion is marginally greater than that of the periphery; however, it does not meet the criteria outlined in the study for "at least two vessels with distinctly faster blood flow." Additionally, the overall blood flow velocity of the lesion remains low, leading to its classification as a low-speed supplying pattern. The color bar corresponds to speed (in mm/s). (**c**) The SR-US direction map displays a complex pattern of diverse and intersecting flow directions, characterized as a mixed direction pattern. The color bar represents blood flow direction (red and blue indicate flow toward and away from the transducer, respectively) and the normalized number of localized microbubbles. Horizontal scale: 5mm. **Supplementary Figure S3**: SR-US parameter maps of an atypical focal nodular hyperplasia. (**a**) The SR-US density map indicates that the microvascular structure of the lesion is abundant and uniformly distributed across the lesion area, presenting a well-distributed density pattern. The color bar corresponds to the normalized number of localized microbubbles. (**b**) The SR-US velocity map indicates that the lesion exhibits a high overall vascular velocity, characterized by a high-speed feeding pattern; however, no central "radial" trunk vessels are discernible. The color bar corresponds to speed (in mm/s). (**c**) The SR-US direction map displays intersecting and overlapping blood flow directions, characterized as a mixed direction pattern. The color bar represents blood flow direction (red and blue indicate flow toward and away from the transducer, respectively) and the normalized number of localized microbubbles. Horizontal scale: 2mm. **Supplementary Figure S4**: SR-US parameter maps of a hepatocellular carcinoma resembling focal nodular hyperplasia. (**a**) The SR-US density map reveals that the microvascular structure of the lesion is somewhat sparse but exhibits a relatively uniform distribution across the lesion area, thus is classified as a well-distributed density pattern. The color bar corresponds to the normalized number of localized microbubbles. (**b**) The SR-US velocity map shows that the central vascular flow velocity of the lesion is comparatively high, presenting a high-speed feeding pattern. The color bar corresponds to speed (in mm/s). (**c**) The SR-US direction map reveals the presence of prominent central vessels exhibiting a centrifugal flow direction, thereby indicating a classification as a centrifugal directional pattern. The color bar represents blood flow direction (red and blue indicate flow toward and away from the transducer, respectively) and the normalized number of localized microbubbles. Horizontal scale: 2mm. **Supplementary Figure S5**: SR-US parameter maps of a hepatocellular carcinoma resembling liver metastasis. (**a**) The SR-US density map reveals that the microvascular structure of the lesion is predominantly located in the peripheral region, with a noticeable absence of microvascular structure in the central area, resulting in a peripheral-distributed density pattern. The color bar corresponds to the normalized number of localized microbubbles. (**b**) The SR-US velocity map shows that the blood flow velocity within the lesion is comparatively high, presenting a high-speed feeding pattern. The color bar corresponds to speed (in mm/s). (**c**) The SR-US direction map indicates that vessels with high velocity exhibit blood flow directed toward the transducer, thereby being categorized as a eccentric direction pattern. The color bar represents blood flow direction (red and blue indicate flow toward and away from the transducer, respectively) and the normalized number of localized microbubbles. Horizontal scale: 5mm

## Data Availability

The data that support the findings of this study are available on request from the corresponding author, Ping Liang, upon reasonable request.

## Abbreviations

CE-PD: Contrast-enhanced power Doppler
CEUS: Contrast-enhanced ultrasound
FLL: Focal liver lesion
FNH: Focal nodular hyperplasia
HCC: Hepatocellular carcinoma
LM: Liver metastasis
SR-US: Super-resolution ultrasound
