# Supplementary material for: Focal liver lesions: multiparametric microvasculature characterization via super-resolution ultrasound imaging
Source: Eur Radiol Exp. 2024 Dec 5;8:138. doi: 10.1186/s41747-024-00540-3 (PMC11621259; doi:10.1186/s41747-024-00540-3)
Supplement: Supplementary file 1 — Additional file 1: Supplementary Table S1 Modified for the ultrafast imaging mode in Resona 7. Supplementary Figure S1: Display the five different manually cross-sectioned ROI of the same SR-US density map with Fig. 4. The vessel size measured as the full width at half-maximum of the amplitude distribution of the Gaussian fit curve on SR-US image and corresponding CE-PD image in each selected ROI. CE-PD = contrast-enhanced power Doppler, SR-US = super-resolution ultrasound, ROI = region of interest. Supplementary Table S2: The cross-section measurement of CE-PD mode and SR-US mode in each selected ROI. Supplementary Figure S2: SR-US parameter maps of an atypical liver metastasis. (a) The SR-US density map indicates that the microvascular structure of the lesion exhibits chaotic distribution throughout the lesion area, displaying an irregularly distributed density pattern. The color bar corresponds to the normalized number of localized microbubbles. (b) The SR-US velocity map indicates that the central vascular velocity of the lesion is marginally greater than that of the periphery; however, it does not meet the criteria outlined in the study for "at least two vessels with distinctly faster blood flow." Additionally, the overall blood flow velocity of the lesion remains low, leading to its classification as a low-speed supplying pattern. The color bar corresponds to speed (in mm/s). (c) The SR-US direction map displays a complex pattern of diverse and intersecting flow directions, characterized as a mixed direction pattern. The color bar represents blood flow direction (red and blue indicate flow toward and away from the transducer, respectively) and the normalized number of localized microbubbles. Horizontal scale: 5mm. Supplementary Figure S3: SR-US parameter maps of an atypical focal nodular hyperplasia. (a) The SR-US density map indicates that the microvascular structure of the lesion is abundant and uniformly distributed across the lesion area, presenting a well [file 41747_2024_540_MOESM1_ESM.pdf]

Focal liver lesions: multiparametric microvasculature characterization via super-resolution ultrasound imaging

ELECTRONIC SUPPLEMENTARY MATERIAL

SR-US imaging settings and data acquisition

A modified clinical ultrasound scanner (Resona 7, Mindray, Shenzhen, China) equipped with a low frequency 192-element convex array transducer (SC6-1U, Mindray, central frequency, 3.1 MHz; bandwidth at -20 dB, 1.2-5 MHz; pitch, 0.335 mm) was used for SR-US imaging. An ultrafast imaging sequence of diverging waves was designed for the convex array transducer, which consisted of 5 inclination angles from −15° to 15°, at 7.5° increments. For each angle, two consecutive cycles of pulses were transmitted at a low frequency of 2.75 MHz to improve penetrability to the deeper tissues. Multiangle coherently compounded and beamformed in-phase/quadrature (IQ) data were recorded to enhance the backscattered echoes, allowing the combined imaging frame rate to reach 350-500 Hz (which depends on the imaging depth and the imaging field of view) and enabling fast data acquisition. SR-US imaging necessitates the adequate accumulation of microbubble signals to comprehensively delineate the microvasculature. Conventional clinical scanners operate at a relatively low imaging frame rate, approximately 10 to 15 Hz, thereby often requiring an extended data acquisition period—ranging from tens of seconds to several minutes—to gather sufficient data. However, prolonged acquisition durations inevitably heighten the susceptibility to motion artifacts. While the ultrafast imaging capability allows for the acquisition of substantial MB signal within few seconds, requiring patients to hold their breath for only a brief duration (~10 seconds). This significantly mitigates the effect of abdominal respiratory motion on SR-US image reconstruction. Detailed modifications specific to ultrafast imaging in the Resona 7 system are provided in Supplementary Table 1.

Supplementary Table S1 Modified for the ultrafast imaging mode in Resona 7

Setting parameter	Conventional clinical scanner	High frame-rate clinical scanner	Clinical applicability
Transmission sequence	Non-Linear Fundamental	Fundamental	• Short acquisition time (~10 s).<br>• Ultrafast ultrasound imaging mitigates tissue motion.<br>• High time resolution improves continuous microbubble tracing performance.
Transmission waveform	Focusing wave	Diverging wave	
Number of transmit angles	1	5	
Inclination angles	0°	−15° to 15°, with a step of 7.5°	
Mechanical index (MI)	0.08	0.2	
Frame-rate (Hz)	low frame rate (around 10 to 15Hz)	High frame rate (around 350 to 500 Hz)	
Acquisition time	Dozens of seconds or even several minutes	About 10 seconds (within a single breath hold)	

SR-US was performed by two radiologists with 8 and 10 years of experience in performing abdominal contrast-enhanced ultrasound (CEUS). Patients were placed in the supine or lateral position to ensure that the target lesion could be maximally visualized was in the middle of the screen without blockage by lung gas or rib shadow. After a bolus injection of 1.5 mL contrast MBs (SonoVue, Bracco, Milan, Italy) followed by flushing with 5 mL of normal saline, 3500-5000 frames consecutive IQ data were recorded from the first 10 seconds (to ensure sufficient MB counts for generating the microvascular architecture[1] and clinical feasibility) of the arterial phase (usually within

30-45 seconds from the time of the injection[2]). The dose of MB was chosen based on the conventional dose for focal liver characterization in the institution using this ultrasound system in CEUS mode. During data acquisition, patients were asked to hold their breath, while the operator held the probe still to significantly reduce motion interference and improve the accuracy of MBs localization and tracking. Subsequently, CEUS was conducted 30 minutes after the initial injection to facilitate verification and comparative analysis. A real-time monitor was employed to ensure the absence of residual MBs from the first injection. The procedure was carried out using the same MB dose and field of view as utilized in the SR-US imaging. A two-minute CEUS video clip was recorded through continuous scanning under conditions of controlled, slow respiration.

SR-US imaging processing

Motion correction

To address motion artefacts, the frame with large motion amplitudes were manually deleted from the collected 10 seconds of raw IQ data, and approximately 2000 stable and continuous frames were selected for motion correction based on interframe correlations. The spatial displacement in the time domain can be converted into phase information in the frequency domain via the Fourier transform[3]. By calculating the phase information of the mutual power spectrum between different frames, the displacement (i.e., tissue motion) between different frames can be estimated and the corresponding affine matrix can be obtained. Then, the motion was compensated by matrix transformation for the following steps to correct the MB localization.

MBs isolation, localization and Doppler processing

To remove the static tissue signal and extract the MBs signal, the singular value decomposition (SVD) based spatio-temporal filtering was performed on the acquired IQ data[4]. The MB signal data were compensated based on the estimated tissue motion. Then, the centroids of the bubble were spatially localized using the weighted average of the MB pixel values[5]. To improve the computational efficiency of the SVD process, the acquired IQ data were divided into multiple datasets (each containing several tens of frames), and SVD-based clutter filtering was performed for each dataset to extract the MB signals. Based on the same MBs datasets, we can also accumulate MB signal power along the temporal dimension to generate contrast-enhanced power Doppler (CE-PD) images. CE-PD provides enhanced contrast over traditional color and power Doppler images, and was quantitatively compared with SR-US to assess image resolution[6].

Tracking and mapping

For MB tracking, a modified Kalman filtering was used to improve the accuracy of the MBs tracking in this high-concentration MB application[7]. In brief, the trajectories were used to predict the nonlinear motion of MBs, and exploits parameters such as acceleration and motion direction to restrict the tracking trajectory. All the MB positions were summed to generate a vessel density map depicting the vessel morphology. Based on each MB trajectory, the instantaneous in-plane velocities of the bubbles can be evaluated to generate SR-US velocity and direction maps. All the SR-US images were reconstructed at a 92.4 μm ((in both the axial and lateral dimensions) pixel resolution.

SR-US quantitative parameters

In this study, the selection of parameters was conducted with careful consideration. Specifically, parameters were chosen based on their morphological and hemodynamic significance, as established in previous research. Furthermore, we were limited to deriving parameters using the algorithms that were feasible and accessible to our team. Vessel tortuosity and vessel distance were excluded from the analysis due to the algorithms for these parameters being highly contingent upon the accurate identification and selection of the target vessel, as well as the current inability to provide a clear definition of a representative tumor vessel[8-14].

Vessel diameter

The amplitude distribution Gaussan fit curve of a blood vessel selected in a region of interest (ROI) was plotted, and the FWHM of the curve was measured and defined as the vessel diameter of the vessel.

Vascular density (VD)

Vascular Density (VD) was defined as the ratio of the number of labelled vessel pixels to the total number of pixels within an ROI region. B-mode images were used to delineate the outer boundary of the total lesion area. By shrinking the radius inwards (the distance between multiple points on the boundary to the center) inward by 50%, each lesion could be divided into central and peripheral regions, the latter defined as the outer 50%; removing the peripheral area yielded the center regions. The total, central and peripheral vessel densities were calculated as follows (1):

$$VD = \frac{Vessel\ Pixels}{Overall\ ROI\ Pixels} \quad (1)$$

For the calculation of liver parenchymal vascular density, the 1 cm diameter circular ROIs were selected at the same depth range as the lesion and at least 1 cm outside the lesion boundary on B-mode images, avoiding visible large main vessels.

Velocity

The blood flow velocity was calculated as the distance travelled by MBs in space per unit time. The velocity in each vessel was extracted for the whole ROI, yielding a region velocity array, which was averaged to obtain the mean velocity. The maximum and minimum detected values in the area were defined as the maximum and minimum velocities, respectively.

Perfusion index (PI)

Perfusion index (PI) was defined as the product of the average blood flow velocity $\overline{V}$ and the VD in a selected ROI and reflects the activity level of the microvasculature. The total, central and peripheral perfusion indices were calculated using formula (2):

$$PI = \overline{V} \times VD \quad (2)$$

Fractal dimension

The fractal dimension of the tumor vasculature was estimated using a box-counting algorithm on the SR-US maps[15]. The tumor vasculature image was covered with grids consisting of boxes, and the number of boxes contained in the vessel (N) was subsequently counted, i.e., the number of boxes covering the vascular structures. Obviously, when the size of the boxes is reduced, the number of boxes required for covering the vasculature increases, or as stated in equation form,$N \propto L^{-D}$, where L is the box size of the grid, and D is the fractal dimension[16]. After conversion, the fractal dimension D is obtained, as shown in formula (3):

$$D = \lim_{L \to 0} \frac{log\,N(L)}{log\frac{1}{L}} \quad (3)$$

By changing the box size L, the numbers of boxes N for different sizes can be obtained, the data are subsequently fitted by a first-order equation to obtain the slope of the corresponding relationship between $\log\frac{1}{L}$ and $\log N(L)$, i.e., the fractal dimension.

Local flow direction entropy

Local flow direction entropy is used to measure the degree of confusion in the direction of blood flow[17]. We calculated the local flow entropy of vessel in a sub-region of 6 mm×6 mm on the SR-US direction maps, as formula (4). Local flow entropy values were averaged over the entire tumor area.

$$H(i,j) = -\sum p(i,j) \log p(i,j) \tag{4}$$

Where p is the probability of flow direction.

SR-US image and quantification of resolution

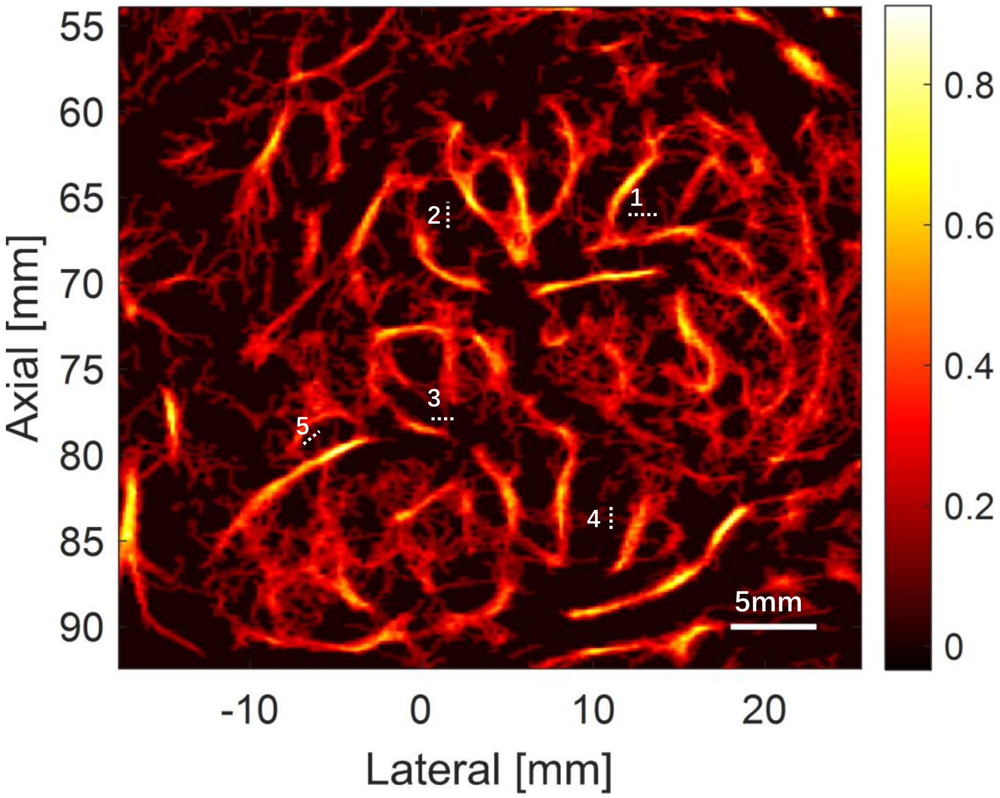

Supplementary Figure S1: Display the five different manually cross-sectioned ROI of the same SR-US density map with Figure 4. The vessel size measured as the full width at half-maximum of the amplitude distribution of the Gaussian fit curve on SR-US image and corresponding CE-PD image in each selected ROI. CE-PD = contrast-enhanced power Doppler, SR-US = super-resolution ultrasound, ROI = region of interest.

Supplementary Table S2: The cross-section measurement of CE-PD mode and SR-US mode in each selected ROI.

ROI Number	CE-PD (μm)	SR-US (μm)
**1**	1032	138
**2**	736	134
**3**	549	91
**4**	687	144
**5**	823	134
**average value**	795.4±160.1	128.4±18.6

Note. —The average value is presented as mean ± standard deviation. CE-PD = contrast-enhanced power Doppler, SR-US = super-resolution ultrasound, ROI = region of interest

SR-US parametric patterns of FLLs

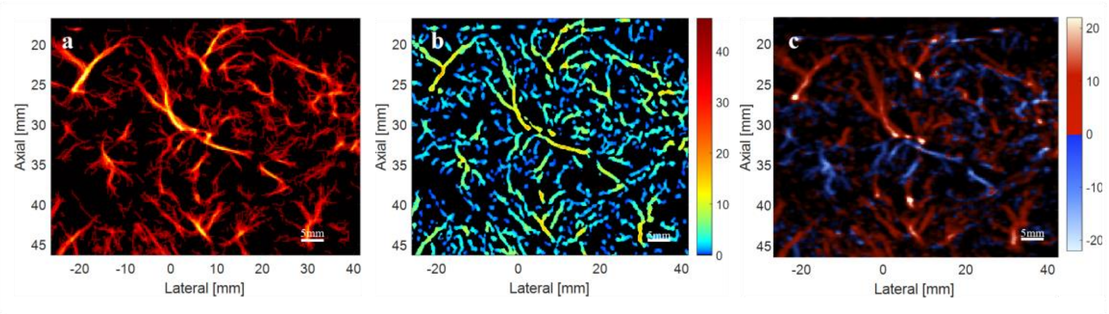

Supplementary Figure S2: SR-US parameter maps of an atypical liver metastasis. (a) The SR-US density map indicates that the microvascular structure of the lesion exhibits chaotic distribution throughout the lesion area, displaying an irregularly distributed density pattern. The color bar corresponds to the normalized number of localized microbubbles. (b) The SR-US velocity map indicates that the central vascular velocity of the lesion is marginally greater than that of the periphery; however, it does not meet the criteria outlined in the study for "at least two vessels with distinctly faster blood flow." Additionally, the overall blood flow velocity of the lesion remains low, leading to its classification as a low-speed supplying pattern. The color bar corresponds to speed (in mm/s). (c) The SR-US direction map displays a complex pattern of diverse and intersecting flow directions, characterized as a mixed direction pattern. The color bar represents blood flow direction (red and blue indicate flow toward and away from the transducer, respectively) and the normalized number of localized microbubbles. Horizontal scale: 5mm.

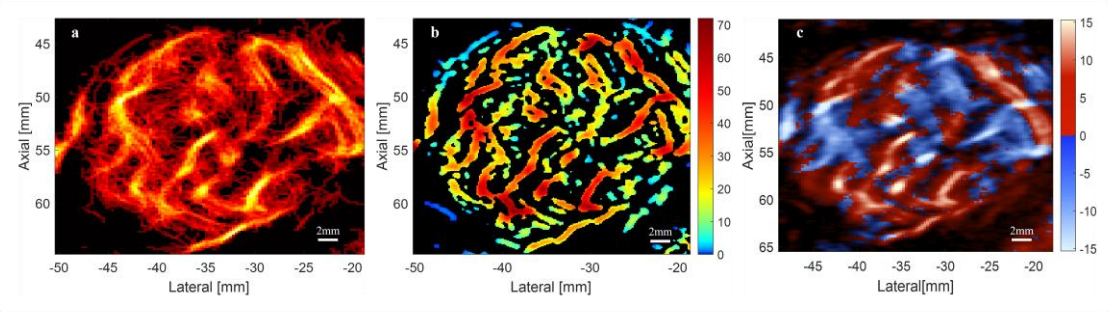

Supplementary Figure S3: SR-US parameter maps of an atypical focal nodular hyperplasia. (a) The SR-US density map indicates that the microvascular structure of the lesion is abundant and uniformly distributed across the lesion area, presenting a well-distributed density pattern. The color bar corresponds to the normalized number of localized microbubbles. (b) The SR-US velocity map indicates that the lesion exhibits a high overall vascular velocity, characterized by a high-speed feeding pattern; however, no central "radial" trunk vessels are discernible. The color bar corresponds to speed (in mm/s). (c) The SR-US direction map displays intersecting and overlapping blood flow directions, characterized as a mixed direction pattern. The color bar represents blood flow direction (red and blue indicate flow toward and away from the transducer, respectively) and the normalized number of localized microbubbles. Horizontal scale: 2mm.

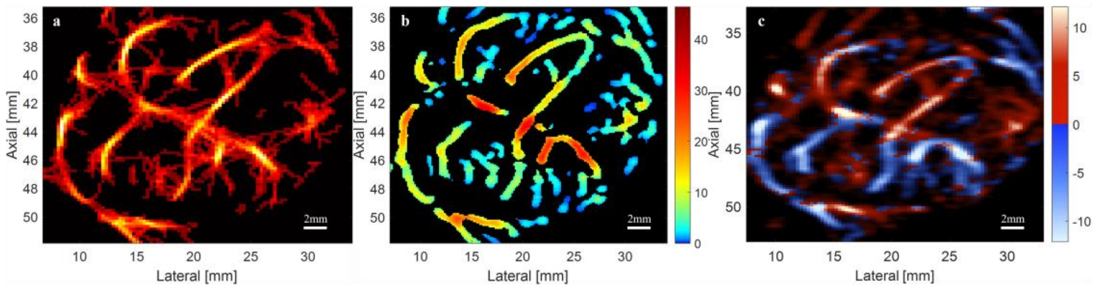

Supplementary Figure S4: SR-US parameter maps of a hepatocellular carcinoma resembling focal nodular hyperplasia. (a) The SR-US density map reveals that the microvascular structure of the lesion is somewhat sparse but exhibits a relatively uniform distribution across the lesion area, thus is classified as a well-distributed density pattern. The color bar corresponds to the normalized number of localized microbubbles. (b) The SR-US velocity map shows that the central vascular flow velocity of the lesion is comparatively high, presenting a high-speed feeding pattern. The color bar corresponds to speed (in mm/s). (c) The SR-US direction map reveals the presence of prominent central vessels exhibiting a centrifugal flow direction, thereby indicating a classification as a centrifugal directional pattern. The color bar represents blood flow direction (red and blue indicate flow toward and away from the transducer, respectively) and the normalized number of localized microbubbles. Horizontal scale: 2mm.

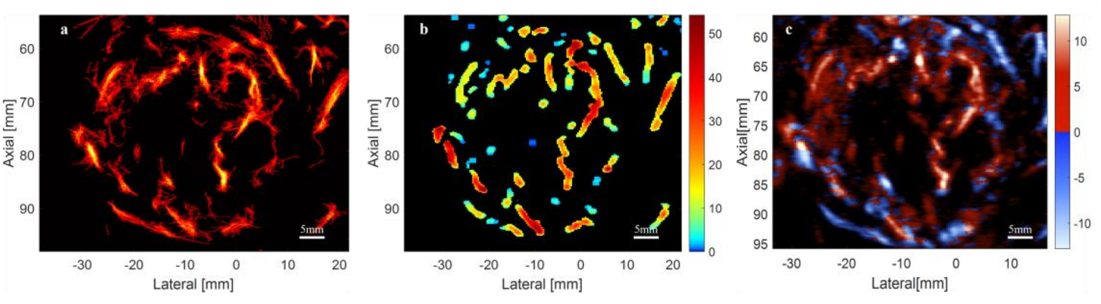

Supplementary Figure S5: SR-US parameter maps of a hepatocellular carcinoma resembling liver metastasis. (a) The SR-US density map reveals that the microvascular structure of the lesion is predominantly located in the peripheral region, with a noticeable absence of microvascular structure in the central area, resulting in a peripheral-distributed density pattern. The color bar corresponds to the normalized number of localized microbubbles. (b) The SR-US velocity map shows that the blood flow velocity within the lesion is comparatively high, presenting a high-speed feeding pattern. The color bar corresponds to speed (in mm/s). (c) The SR-US direction map indicates that vessels with high velocity exhibit blood flow directed towards the transducer, thereby being categorized as a eccentric direction pattern. The color bar represents blood flow direction (red and blue indicate flow toward and away from the transducer, respectively) and the normalized number of localized microbubbles. Horizontal scale: 5mm.

Supplementary References

1 Song P, Rubin JM, Lowerison MR (2023) Super-resolution ultrasound microvascular imaging: Is it ready for clinical use? Z Med Phys 33:309-323

2 Dietrich CF, Nolsøe CP, Barr RG et al (2020) Guidelines and Good Clinical Practice Recommendations for Contrast Enhanced Ultrasound (CEUS) in the Liver - Update 2020 - WFUMB in Cooperation with EFSUMB, AFSUMB, AIUM, and FLAUS. Ultraschall Med 41:562-585

3 Foroosh H, Zerubia JB, Berthod M (2002) Extension of phase correlation to subpixel registration. IEEE Trans Image Process 11:188-200

4 Brown J, Christensen-Jeffries K, Harput S et al (2019) Investigation of Microbubble Detection Methods for Super-Resolution Imaging of Microvasculature. IEEE Trans Ultrason Ferroelectr Freq Control 66:676-691

5 Errico C, Pierre J, Pezet S et al (2015) Ultrafast ultrasound localization microscopy for deep super-resolution vascular imaging. Nature 527:499-502

6 Huang C, Zhang W, Gong P et al (2021) Super-resolution ultrasound localization microscopy based on a high frame-rate clinical ultrasound scanner: an in-human feasibility study. Phys Med Biol 66

7 Tang S, Song P, Trzasko JD et al (2020) Kalman Filter-Based Microbubble Tracking for Robust Super-Resolution Ultrasound Microvessel Imaging. IEEE Trans Ultrason Ferroelectr Freq Control 67:1738-1751

8 Chen Q, Yu J, Rush BM, Stocker SD, Tan RJ, Kim K (2020) Ultrasound super-resolution imaging provides a noninvasive assessment of renal microvasculature changes during mouse acute kidney injury. Kidney Int 98:355-365

9 Demené C, Robin J, Dizeux A et al (2021) Transcranial ultrafast ultrasound localization microscopy of brain vasculature in patients. Nat Biomed Eng 5:219-228

10 Lin F, Shelton SE, Espíndola D, Rojas JD, Pinton G, Dayton PA (2017) 3-D ultrasound localization microscopy for identifying microvascular morphology features of tumor angiogenesis at a resolution beyond the diffraction limit of conventional ultrasound. Theranostics 7:196-204-204

11 Lowerison M, Zhang W, Chen X, Fan T, Song P (2022) Characterization of Anti-Angiogenic Chemo-Sensitization via Longitudinal Ultrasound Localization Microscopy in Colorectal Carcinoma Tumor Xenografts. IEEE Transactions on Biomedical Engineering, Biomedical Engineering, IEEE Transactions on, IEEE Trans Biomed Eng 69:1449-1460

12 Lowerison MR, Huang C, Lucien F, Chen S, Song P (2020) Ultrasound localization microscopy of renal tumor xenografts in chicken embryo is correlated to hypoxia. Sci Rep 10:1-13

13 Lowerison MR, Sekaran NVC, Zhang W et al (2022) Aging-related cerebral microvascular changes visualized using ultrasound localization microscopy in the living mouse. Sci Rep 12:1-11

14 Tatjana O, Stefanie D, Benjamin T et al (2018) Motion model ultrasound localization microscopy for preclinical and clinical multiparametric tumor characterization. Nat Commun 9:1-13

15 Lowerison M, Zhang W, Chen X, Fan T, Song P (2022) Characterization of Anti-

Angiogenic Chemo-Sensitization via Longitudinal Ultrasound Localization Microscopy in Colorectal Carcinoma Tumor Xenografts. IEEE Trans Biomed Eng 69:1449-1460
16 Baish JW, Jain RK (2000) Fractals and cancer. Cancer Res 60:3683-3688
17 Zhu J, Zhang C, Christensen-Jeffries K et al (2022) Super-Resolution Ultrasound Localization Microscopy of Microvascular Structure and Flow for Distinguishing Metastatic Lymph Nodes - An Initial Human Study. Ultraschall Med 43:592-598
